# Evaluation of Plant Origin Essential Oils as Herbal Biocides for the Protection of Caves Belonging to Natural and Cultural Heritage Sites

**DOI:** 10.3390/microorganisms9091836

**Published:** 2021-08-30

**Authors:** Anthoula A. Argyri, Agapi I. Doulgeraki, Eftychia G. Varla, Vasiliki C. Bikouli, Pantelis I. Natskoulis, Serkos A. Haroutounian, Georgios A. Moulas, Chrysoula C. Tassou, Nikos G. Chorianopoulos

**Affiliations:** 1 Institute of Technology of Agricultural Products, Hellenic Agricultural Organisation—DIMITRA, Sofokli Venizelou 1, Lycovrissi, 14123 Attica, Greece; adoulgeraki@aua.gr (A.I.D.); houla94@hotmail.com (E.G.V.); mpvicky@otenet.gr (V.C.B.); p.natskoulis@gmail.com (P.I.N.); ctassou@nagref.gr (C.C.T.); 2 Laboratory of Nutritional Physiology and Feeding, Department of Animal Science Agricultural University of Athens, Iera Odos 75, 11855 Athens, Greece; sehar@aua.gr; 3 Moulas Scientific, Messinias 14, 15234 Chalandri, Greece; gmoulas@moulasscientific.com

**Keywords:** caves, natural biocides, essential oils, MIC, NIC

## Abstract

The present study concerns the serious issue of biodeterioration of the caves belonging to natural and cultural heritage sites due to the development of various microorganisms. Thus, a series of 18 essential oils (EOs) extracted from various Greek plants were evaluated in vitro (concentrations of 0.1, 0.2, 0.5, 1.0 and 5.0% *v*/*v*) against 35 bacterial and 31 fungi isolates (isolated from a Greek cave) and the antimicrobial activity was evident through the changes in optical density of microbial suspensions. In continuance, eight (8) representative bacterial and fungal isolates were further used to evaluate the minimum inhibitory concentration (MIC) and non-inhibitory concentration (NIC) values of the most effective EOs. According to the results, two EOs of *Origanum vulgare* were the most effective by inhibiting the growth of all the tested microorganisms at 0.1% (*v*/*v*), followed by that of *Satureja thymbra* which inhibited all bacterial isolates at 0.1% (*v*/*v*) and fungal isolates at 0.1, 0.2 and 0.5% (*v*/*v*) (depending on the isolate). The MIC ranged between 0.015–0.157 and 0.013–0.156 (*v*/*v*) for the bacterial and fungal isolates respectively, depending on the case. The current study demonstrated that conventional biocides may be replaced by herbal biocides with significant prospects for commercial exploitation.

## 1. Introduction

Caves are usually oligotrophic environments with relatively stable environmental parameters (total darkness or low level of light, low stable temperature and high humidity), but with a wide range of microorganisms that can survive and grow in such conditions [1,2,3]. However, the touristic exploitation of the caves may result to changes in the microclimatic conditions with parallel import of organic matter by the visitors. These factors can majorly affect the microbial composition and proliferation with adverse consequences on the cave rocks and prehistoric paintings [4,5,6,7,8].

The microbial community of caves that has been widely studied, may be formed by bacteria, cyanobacteria, algae and fungi and is usually characterized by high diversity [2,5,9,10,11,12]. Regarding the bacterial community of caves, it is usually constituted by Actinobacteria, Proteobacteria, Bacteroidetes, Firmicutes, Acidobacteria, Planctomycetes, Chloroflexi, Gemmatimonadetes and Cyanobacteria [8,9,12,13]. On the other hand, Ascomycota, Zygomycota, Basidiomycota, Mycetozoa, and Oomycota are the most frequently reported fungal groups in hypogean enviroments [10,13,14,15].

Cave microbiota are metabolically versatile and are involved in important lithogenic processes such as speleothem deposition, but also in unwanted changes such as color covering spots and litholitic processes such as bioweathering of rocks and minerals [16,17,18,19]. Any biological activity that affects the appearance and integrity of the materials is known as “biodeterioration” [20,21]. Previous studies have reported the presence of different bacteria in caves with biodeterioration signs. Some members of the Proteobacteria group are associated with the deterioration of Palaeolithic paintings in Altamira Cave, where it was found to be the dominant metabolically active bacterial group in white, yellow and gray colonizations [22,23]. Zimmermann et al. [24] (using Acidobacteria-specific primers), detected a high diversity of Acidobacteria in Altamira Cave on some pigmented areas (green and black). Alonso et al. [25] have extensively studied the microbial community on black stains formed on the rock surfaces of Lascaux Cave and found that members of Proteobacteria, Actinobacteria, Bacteroidetes, Chloroflexi and Planctomycetes and members of Ascomycota (Dothideomycetes, Eurotiomycetes, Sordariomycetes, Chaetothyriomycetes) consisted the main bacterial and fungal community on these pigmented areas. They mentioned that the occurrence of black stains most likely results from spatially localized conditions promoting particular development or physiology of pigmented microorganisms. Other authors have also reported the presence of stain-forming fungi of Ascomycota (Dothideomycetes, Eurotiomycetes, Sordariomycetes) on black or green wall stains [21,26,27], while cave fungi in general can produce all types of pigments (grey, orange, purple, red, white, yellow) [27,28,29]. Additionally, green, brown or black spots have been attributed to the presence of unwanted photosynthetic microorganisms (Lampenflora) of which the growth has been promoted due to the introduction of lighting into the cave ecosystem for visiting reasons [13].

However, besides the aesthetic appearance, bacteria may cause damage to prehistoric paintings by altering the mineral structure of the rock, since it was found able to produce inorganic and organic acids, neutral and acidic sugars that may degrade rock, speleothems and prehistoric paintings [13,30]. In general, inorganic acids are exclusively produced by bacteria, with some Firmicutes (*Bacillus*) being involved in calcite precipitation in caves which can induce damage in parietal markings (such as prehistoric human markings) [9,27,31]. The mineralization and precipitation of crystals by bacteria is initialized by locally changing the microenvironmental conditions (e.g., by increasing alkalinity supersaturation of CaCO_3_ in aqueous phase). After initial mineralization, the size of small fibre calcite crystals increase gradually, irrespective of metabolic activity of bacteria which may decrease or completely stop. Additionally, extracellular polymeric substances widely produced by bacteria can enhance diffusion gradients, provide nucleation sites and thus facilitate precipitation of minerals [32].

In their turn, fungi can affect rocks through mechanical and biochemical activities [27]. The biomechanical impact of fungi on rocks is due to the penetration by the fungal hyphae into the mineral substrates and by tunnelling into otherwise intact mineral material, for example along crystal planes in sandstone, calcitic, and dolomitic rocks [28,29]. On the other hand, the fungi metabolites weather the substrate (biochemical impact) by changing the pH of the microenvironment or changing the energy states of ions in the rock matrix at its surface. Fungi may excrete organic acids and many other metabolites with metal complexing properties, such as amino acids and phenolic compounds, resulting even in bioconversion of coal, mineral solubilization and accumulation of metals [28,29].

The biodeterioration phenomenon in show caves is usually addressed by applying different types of biocides aiming to microbial decontamination and proliferation, which is the main cause of the problem. Conventional biocides used so far in caves include formaldehyde, benzalkonium chloride (BAC), streptomycin and polymyxin, sodium hypochlorite (NaOCl), 2-octyl-3-isothiazolinone (OIT), mixtures of quaternary ammonium compounds and other chemicals [15,26,33,34,35]. Some drawbacks of the usually applied chemical biocides are the wall and mineral structures degradation (e.g., sodium hypochlorite), utilization of the chemicals (e.g., quaternary ammonium compounds) by bacteria as a sole source of carbon and nitrogen resulting in secondary microbial outbreak, the environmental toxicity effects of certain chemicals in addition to their low efficiency over time [1,13,36,37]. The biocides thymol, silver nitrate and copper sulphate were also tested in different concentrations against bacterial isolates from Magura cave [38]. Although the silver nitrate and copper sulphate showed antimicrobial activity in lower concentrations, the authors proposed thymol as a transparent and colorless compound that did not damage additionally the cave paintings. Herbal biocides including essential oils (EOs) could be considered as potential ‘natural’ biocides alternative to the chemical ones. Except from the aforementioned work of Iliev et al. [38], no studies to our knowledge report treatment of cave microbiota with herbal biocides. In particular, studies including evaluation of EOs or their derivatives have been evaluated for cultural heritage conservation are limited and include studies mainly on over-ground cultural heritage objects [39,40], mural paintings [41,42,43] or mosaic tesserae [44].

The aim of the current study was to evaluate the antimicrobial activity of a series of eighteen (18) essential oils extracted from various Greek plants against 35 bacterial and 31 fungal isolates (previously isolated from Petralona cave) and selection of the three most promising EOs for further evaluation of the MIC and NIC concentration.

## 2. Materials and Methods

### 2.1. Samples Collection and Isolation of Microorganisms

The microorganisms tested in this study were previously isolated from Petralona Cave, Halkidiki, Greece while the cave was closed for maintenance reasons in February 2018. The samplings were performed by trained personnel on cave walls covered by dark stains, by applying swab HyServe™ on a surface of 10 cm^2^ and by detaching small weathered pieces of rocks from the cave walls which were placed in sterilized 2 mL tubes. The samples were then transferred under refrigeration to the laboratory and analyzed for the isolation of cave microbiota.

The following methods were applied to isolate the microorganisms: (a) following serial dilutions by the swab supernatant and spreading 100 μL on the corresponding agar plates, (b) streaking the swabs on the agar media, (c) streaking and placing directly the small weathered rocks on the surface of the agar plates. The agar media that were used for the microbiota isolation were: Plate Count Agar (PCA, LAB149, LabM, Lancashire, UK) for total viable counts, Rose Bengal Chloramphenicol Agar (RBC Agar, BK151HA, Biokar Diagnostics, Cedex, France) for yeasts/moulds, and BG11 agar [45] for cyanobacteria. The PCA and RBC inoculated plates were incubated at 25 °C for up to 10 days, while in the meantime isolation was performed on fully grown colonies. The BG11 inoculated plates were incubated at 25 °C under light provided by fluorescent lamps, however no growth was observed on these media. This standard incubation temperature, higher than air temperature of the Petralona cave (17 ± 1 °C), was chosen (i) according to the media specifications, (ii) as the majority of microorganisms grow faster at 25 °C, and (iii) to compare the results with other cave studies, with similar culture conditions [15,27].

Τhe colonies were transferred and purified on PCA and Yeast Peptone Dextrose agar (YPD, LAB176, LabM, Lancashire, UK) for suspected bacteria and yeasts, respectively and on Malt Extract Agar (MEA, LAB37, LabM, Lancashire, UK) for suspected fungi and incubated for up to 7 days at 25 °C. Oxidase and catalase reaction, colony morphology, Gram stain and cell morphology were applied for suspected bacteria and yeasts, however all the isolates were found to be bacteria. Also, all the isolates from RBC were found to be fungi or bacteria and no yeasts were observed. The purified bacteria were stored in Tryptic Soy Broth with 30% *v*/*v* glycerol (TSB, LabM, LAB004), while the fungi were stored in glycerol solution 70% *v*/*v* and both stored at −80 °C until use. Czapeck yeast-extract agar (CYA) and Glycerol 25% Nitrate Agar (G25N) media were also used in parallel with MEA to differentiate/group the different fungal isolates according to morphology features [15,27,46,47]. The latter methodologies in combination with the resistance in the different EOs tested (Section 2.2) were applied to selected representative isolates of the same phenotype for molecular characterization.

### 2.2. Molecular Characterization

#### 2.2.1. DNA Extraction

Pure culture cells were collected with a microbiological loop (10 μL) from each agar medium (PCA for bacteria, MEA for fungi) and resuspended with 100 μL 10 mM Tris-HCl (pH = 7.6) 1M NaCl. Subsequently, they were heated at 100 °C for 20 min in thermoblock and centrifigurated (10,000 rpm) for 10 min at 4 °C. The supernatant was stored at −20 °C for further use.

#### 2.2.2. PCR Amplification

PCR amplification was conducted according to the method suggested by Doulgeraki et al. [48]. P1–P4 primers for the bacteria and ITS1-ITS4 for the fungi were used for the amplification of the 16S rRNA and ITS respectively. PCR amplifications were made in a final volume of 25 µL of solution containing 400 mM MgCl2, 200 mM dNTPs, 0.2 mM primer, 1 U of thermostable (Taq) DNA polymerase (New England Biolabs, Ipswich, MA, USA), 0.1 mM MgCl2 Buffer (NEB) and 100 ng of DNA template. PCR reaction for bacteria included an initial denaturation step for 10 min at 94 °C, followed by 35 cycles (denaturation at 94 °C for 1 min, primer annealing at 40 °C for 1 min and extension at 68 °C for 2 min) and a final extension step at 68 °C for 10 min. On the other hand, PCR reactions for fungi included an initial denaturation step at 94 °C for 3 min, followed by 35 cycles (denaturation at 94 °C for 30 sec, primer annealing at 52 °C for 30 sec and extension at 68 °C for 2 min) and a final extension step at 68 °C for 10 min.

#### 2.2.3. Electrophoresis, Quantification and Purification of PCR Products

Aliquots (4 µL) of PCR products were further processed with gel electrophoresis (100 V) in 1.5 % (*w*/*v*) agarose gel using GelRed (Biotium) staining method for confirmation of the success of the PCR procedure applied. The DNA bands were visualized using UV illumination via Gel-Doc system (Biorad, Hercules, CA, USA). Following that, 20 μL of PCR product was purified with solutions of sodium acetate (3M) and ethanol according to [49]. The pellet was resuspended in 20 μL dd H_2_O. The DNA concentrations were quantified through Nanodrop™ 2000 (Thermo Scientific, Walthman, MA, USA).

#### 2.2.4. Sequencing of Isolates

The sequencing took place at CEMIA (Department of Immunology and Histocompatibility of the University of Thessaly), based on the sequence of the V1–V3 part of 16S rRNA for the bacteria and ITS for the fungi. The sequencing results were compared with the available results on the NCBI data base (https://blast.ncbi.nlm.nih.gov/Blast.cgi. (accessed on 30 June 2021) ) [50].

### 2.3. Essential Oils

The essential oils used in the current study are reported in Table 1. The initial sampling was performed through the selection of the appropriate plants of *Salvia* (A), *Foeniculum* (B), *Satureja* (C), *Juniperus* (D, E, F, M), *Citrus* (G, H, I, K, L, N, O), *Laurus* (P), *Origanum* (Q, R), as they are referred in Table 1 [51,52,53,54]. From 2016 to 2018 Greek indigenous aromatic herbs, shrubs and trees were collected from various locations and habitats of Greece. Collection details of all herbal taxa are included in Table 1. A voucher specimen for each plant sampled is deposited in the herbarium of the Agricultural University of Athens, Athens, Greece. The EO of *O. vulgare* (Q) originated from a wild oregano plant, while the EO of *O. vulgare* (R) originated from a cultivated oregano plant. *Origanum vulgare* (R) EO was kindly provided by Ecopharm Hellas S.A. (Macedonia, Greece). With regards to G, H, I, J, K and L (Table 1), the original material of the study consisted of the cold pressed essential oils (CPEOs) derived from the industrial processing of four different Citrus species, which were kindly provided by the fruit juice industry Christodoulou Bros SA [53]. All EOs were obtained by hydro-distillation, according to previously described procedure [51,52,53,54].

### 2.4. Preparation of the Inocula

#### 2.4.1. Preparation of the Bacterial Inocula

The strains were revived from a stock culture stored at −80 °C, by inoculating 100 μL of the culture to 10 mL Brain Heart Infusion broth (BHI, LabM, LAB049), followed by incubation at 25 °C for 1 to 5 days (depending on the isolate). A subculture was prepared by inoculating 100 μL of the 1st culture in 10 mL of fresh BHI, followed by incubation at 25 °C for 1 to 3 days (depending on the isolate) [51,55]. For the inoculum preparation, each bacterial culture was serially diluted in ¼ strength Ringer solution to achieve a final concentration of ca. 10^4^ CFU/mL in each microplate well.

#### 2.4.2. Preparation of the Fungal Inocula

Fungal cultures were prepared by inoculating MEA agar plates with the fungal cultures stored at −80 °C, followed by incubation at 25 °C for 7 days [56,57]. Spore suspensions were prepared by pouring sterile saline solution (0.85% NaCl) with 0.05% (*v*/*v*) Tween80 (Merck, Darmstadt, Germany) in the Petri dish and scratching the mycelium surface with a sterile triangle cell spreader. The resulting suspension was filtered through sterile gauze to remove mycelium structures and spore clumps. The filtrated suspension was serially diluted and the final concentration of spores was assessed by a haemocytometer. 100 μL of the appropriate spore dilution were inoculated in bijoux bottles with 20 mL of Yeast Extract Sucrose semi-solid agar (YES: yeast extract 20 g; sucrose 150 g; MgSO_4_·7H_2_O 0.5 g; LabM Technical Agar No3 1.22 g; distilled water up to 1000 mL) to achieve a final concentration of ca. 10^3^ spores/mL in each microplate well. The application of YES semi-solid agar for fungal kinetic studies with turbidimetric measurements has been reported as an accurate and universal choice, independently of the colour and size of conidia, not only due to its transparency, but also for its ingredients offering simple preparation and homogeneous result [57,58].

### 2.5. Screening of the 18 EOs Antimicrobial Activity

Τhe 18 EOs antimicrobial activity was tested in vitro in 96-well-plates in various concentrations against the isolated bacteria and fungi by measuring the optical density (OD) changes (610 nm) in a microplate reader apparatus (Versamax^TM^). Each EO was diluted to DMSO solution to prepare the appropriate stock solutions. Sterile 96-well flat-bottom polystyrene microplates were used for the experiments and 160 μL of BHI broth, 20 μL of the appropriate EO stock solution and 20 μL of bacterial or fungal inoculum were added in each well to achieve final concentrations of 0.1, 0.2, 0.5, 1 and 5% *v*/*v* of EO. Wells without the addition of the respective EO were used as positive controls, by inoculating 20 μL of each inoculum or as negative controls by inoculating 20 μL of ¼ strength Ringer solution. The microplates were consequently sealed with highly transparent membranes to avoid contamination, incubated for up to 4 days at 25 °C at 300 rpm using an orbital shaker (RSLAB-7) and the absorbance measurements were recorded every 24 h.

### 2.6. Determination of Minimum Inhibitory Concentration (MIC) and Non-Inhibitory Concentration (NIC) of the Most Effective EOs

Eight (8) representative bacterial (*Bacillus* sp. R1P4, *Stenotrophomonas* sp. S2P9, *Paenibacillus* sp. R2P2 and *Paenibacillus* sp. BP6) and fungal (*Fusarium* sp. R2R11, *Penicillium* sp. R1R6, *Clonostachys* sp. S1R1 and *Cladosporium* sp. BP1) isolates were further used to evaluated the MIC of the three most effective EOs i.e., *Origanum vulgare*—wild (Q), *Origanum vulgare* (R) and *Satureja thymbra* (C).

160 μL of BHI broth (for bacteria) or YES semi-solid agar (for fungi) were mixed with 20 μL of the appropriate EO stock solution (DMSO solution) and the resulting 180μL aliquots of growth medium mixed with different concentrations of each essential oil were transferred to the wells of a 96-well microplate. Two (2) fold dilutions were developed by transferring a constant volume of 180 μL from column to column. 20 μL of bacterial or fungal inoculum were then added in each well to achieve final concentrations of 0.5%, 0.4%, 0.3%, 0.25%, 0.2%, 0.15%, 0.125%, 0.1%, 0.075%, 0.0625%, 0.05%, 0.0375%, 0.03125%, 0.025%, 0.01875%, 0.015625%, 0.0125%, 0.009375%, 0.0078125%, 0.00625%, 0.00390625% and 0.003125% *v*/*v* of EO and ca. 10^4^ CFU/mL for bacteria [34,35] or ca. 10^3^ spores/mL for fungi [36] in each microplate well. Consequently, each microplate was sealed with highly transparent membranes to avoid contamination and incubated in a microplate reader (Versamax TM) at 25 °C, while OD measurements (610 nm) were carried out every 10 min for 48 h for bacteria and every 20 min for 96 h for fungi. Growth media (BHI or YES) with no inoculum and inoculated media with no essential oil were used as controls.

### 2.7. MIC and NIC Calculation

The effect of the growth, measured by the OD method, is manifested by a reduction in the area under the OD/time curve relative to a control well at any specified time. By calculating the area using the trapezoidal rule, the relative amount of growth was obtained using the ratio of the test area to that of the control, termed the fractional area *(fa*) [59]. MIC and NIC determinations were performed according to previously reported method [55,58,60], which represent a modification of the primary Lambert-Pearson model (LPM) [59]. This method refers to the use of Gompertz function to fit the observations and produce a dose response profile and has been updated by Lambert and Lambert [60] in the form of Equation (1):

(1) $$fa=\exp\left[-{\left(\frac{x}{P_{1}}\right)}^{P_{2}}\right]$$

*fa* represents the fractional area, *x* is the inhibitor concentration (mg/L), *P*_1_ is the concentration at maximum slope (of a log *x* vs. *fa* plot) and *P*_2_ is a slope parameter. The MIC of each single substance may be defined as the intercept of the concentration axis to the tangent at the maximum gradient of the *fa*/log concentration curve. Thus, from the LPM may be formulated the following equation:

(2) $$MIC=P_{1}\exp\left(\frac{1}{P_{2}}\right)$$

The non-inhibitory concentration is the concentration below which normal visible growth was observed, this was defined as the intercept of the tangent at the maximum gradient of the fa/log concentration curve to the *fa* = 1 contour. From the LPM this can be formulated as:

(3) $$NIC=P_{1}\exp\left(\frac{1-e}{P_{2}}\right)$$

where *e* is the value of the exponential of 1 (approx. 2.718).

Data were fitted to the LPM using non-linear least squares regression analysis assuming equal variance. In addition, the MIC and NIC values were estimated with statistical package Fig.P 2.5. 

## 3. Results and Discussion

### 3.1. Microorganisms

According to the results of the molecular characterization, the isolated bacteria belonged to the Firmicutes (16 isolates), Proteobacteria (15 isolates) and Actinobacteria (4 isolates) (Table 2, Supplementary Table S1). Previous culture dependent and culture independent studies in caves have also reported the frequent presence of the phyla recorded in this study [2,9,61]. *Bacillus* (12 isolates) was the most frequently identified genus among bacteria, followed by *Achromobacter* (9)*, Paenibacillus* (4)*, Rhodococcus* (4)*, Sinorhizobium* (4) and *Stenotrophomonas* (2). *Bacillus* has been frequently isolated in show caves as well as in non-visited caves and their abundance in the cave ecosystems is probably due to the dispersal of their bacterial spores [9,11,19,61,62,63]. Previous studies have also reported the presence of *Achromobacter* [26,37,61]. *Paenibacillus* [19,61,62,64,65], *Rhodococcus* [19,61,64], *Sinorhizobium* [62] and *Stenotrophomonas* [37,61,62] in caves across the world.

All the isolated fungi of the study belonged to the phylum Ascomycota and more specifically in the classes Sordariomycetes (11 isolates), Eurotiomycetes (7 isolates) and Dothideomycetes (1 isolate) (Table 3, Supplementary Table S2). Ascomycota is usually the dominating phylum along the cave mycobiota, with Zygomycota and Basidiomycota being less present [4,13,14,15,18,66,67,68,69]. *Penicillium* (9 isolates) was the most apparent genus followed by *Clonostachys* (5), *Fusarium* (4), *Doratomyces* (4), *Cephalotrichum* (3), *Talaromyces* (2), *Acremonium* (2), *Xenoacremonium* (2), *Trichurus* (1) and *Cladosporium* (1). *Penicillium* [4,15,18,21,29,66,67,68,69,70,71,72,73,74] and *Fusarium* [4,18,21,29,66,72,74] are two of the most reported fungi present in caves. *Clonostachys* [21,29,68,72,74], *Doratomyces* [21,66,67,68,71,72], *Cephalotrichum* [21,29,68,69,74], *Talaromyces* [21,68,69,72,73,74], *Acremonium* [15,18,21,29,66,67,68,69,72,75,76], *Xenoacremonium* [68], *Trichurus* [70,71] and *Cladosporium* [4,15,18,21,29,66,67,68,69,70,72,74,75], were also identified in previous studies.

In this study, the retrieval of cyanobacteria or yeasts was not feasible from the specific spots of the cave walls covered by dark stains (either by swab sampling or by small weathered pieces of rocks). This was maybe due to the fact that the specific spots of epilithic biofilm or surface rock pieces that was studied was either not colonized by cyanobacteria and yeasts, or the cyanobacteria and yeasts present in this case were in very low numbers or non-culturable (in general or on this specific culture conditions). Indeed, it is reported previously that cyanobacteria or yeasts in some caves may be present in lower numbers than the other microbiota or are non-culturable [14,77,78].

### 3.2. Antimicrobial Activity of the 18 EOs

Regarding the effect of the different EOs against the bacterial and fungal microbial groups, besides the 3 most effective EOs (*O. vulgare* Q and R and *S. thymbra* C), a diversity was observed in the resistance against the rest EOs between different isolates and between isolates of the same genus. More specifically, all bacterial isolates were inhibited at a concentration of 0.1% of the EOs *O. vulgare* (Q), *O. vulgare* (R) and *S. thymbra* (C) (Table 2). The majority of the *Bacillus* isolates (12 in total), exhibited resistance against all the remaining EOs [other than *O. vulgare* (Q and R) and *S. thymbra* (C)]. Two (2) *Bacillus* isolates showed sensitivity at *C. limon* (Ν) 1% and 1 isolate showed sensitivity in the majority of the EOs tested (Table 2). The *Achromobacter* isolates (9 in total) were in majority inhibited by *C. limon* (Ν) 1% (7 isolates) and *C. limon* (Κ) 1% (1 isolate), while some were inhibited by *L. nobilis* (P) 1% (3 isolates) and *F. vulgare* (Β) 1% (2 isolates). The *Paenibacillus* isolates (4) were also inhibited by several EOs (Table 2) and all isolates showed different phenotypes (inhibited by different EOs). All *Rhodococcus* (4 isolates) showed sensitivity at *L. nobilis* (P) 1%*,* while 1 of them was additionally inhibited by *C. limon* (Ν) 1%. All *Shinorhizobium* (4 isolates) exhibited sensitivity at *C. limon* (Ν) 1% and *L. nobilis* (P) (2 isolates at 1% and 2 at 0.5%), and 1 to *F. vulgare* (Β) 1%. One (1) of the 2 *Stenotrophomonas* isolates showed sensitivity against *C. limon* (Ν) and *L. nobilis* (P) 1% (Table 2).

Regarding the fungi, all isolates were inhibited at a concentration of 0.1% *O. vulgare* (Q) and (R) (Table 3). The *Penicillium* genus (9 isolates in total), showed high diversity in the sensitivity between the different EOs (Table 3), exhibiting sensitivity in *S. thymbra* (C) 0.1, 0.2 or 0.5 % (9 isolates), *L. nobilis* (P) 0.5% (6 isolates), *S. triloba* (A) and *F. vulgare* (Β) 1% (3 isolates), *C. limon* (Ν) 1% (1 isolate) and *C. aurantium* 1% (1 isolate). *Clonostachys* genus (5 isolates) exhibited sensitivity against *S. thymbra* 0.2% and *C. limon* (Ν) 1%, while 1 isolate was additionally inhibited by *L. nobilis* (P) 1%. The *Fusarium* isolates (4) were inhibited by *S. thymbra* (C) 0.5%, while 1 isolate was additionally inhibited by *L. nobilis* (P) 0.5%. All the *Doratomyces* isolates (4), were inhibited by *S. thymbra* (C) 0.2%, *C. limon* (Ν) 1%, *C. reticulata* (Κ) 1% and *L. nobilis* (P) 0.5%. All *Cephalotrichum* isolates (3), showed sensitivity against *S. thymbra* 0.1%, *L. nobilis* (P) 0.5%, *C. limon* (Ν), *C. aurantium* (O), *S. triloba* (A) and *F. vulgare* (Β) 1%, while 2 of them were additionally inhibited by *C. reticulata* (Κ) and *J. drupacea* (F) 1% and 1 of them was additionally inhibited by *J. drupacea* (Ε). The 2 *Talaromyces* isolates were inhibited by *S. thymbra* (C) 0.2% and *L. nobilis* (P) 0.5%. Finally, the rest of the singleton isolates also showed diversity in antimicrobial resistance (Table 3).

According to the above, the most effective EOs were *Origanum vulgare* wild (Q) and *Origanum vulgare* (R) that inhibited the growth of all the tested bacterial and fungal microbial groups in a concentration of 0.1% (*v*/*v*), followed by *Satureja thymbra* (C) which inhibited all bacterial isolates in concentration of 0.1% and fungal isolates at 0.1, 0.2 and 0.5% (*v*/*v*) (depending on the isolate) (Table 4). The next EO with medium to low antimicrobial activity (0.5–1.0% *v*/*v*) against 37 microorganisms was that of *Laurus nobilis* (P), followed by *Citrus limon* (Ν) EO which showed low antimicrobial activity (1% *v*/*v*) against 32 microorganisms (Table 4). The results of the current study, regarding the antimicrobial activity of the natural biocides, are in accordance with previous studies relevant to the in vitro action of essential oils and their components against various bacteria and fungi that occurred in food systems [79,80].

### 3.3. MIC and NIC of the Most Effective EOs

**Figure 1:**
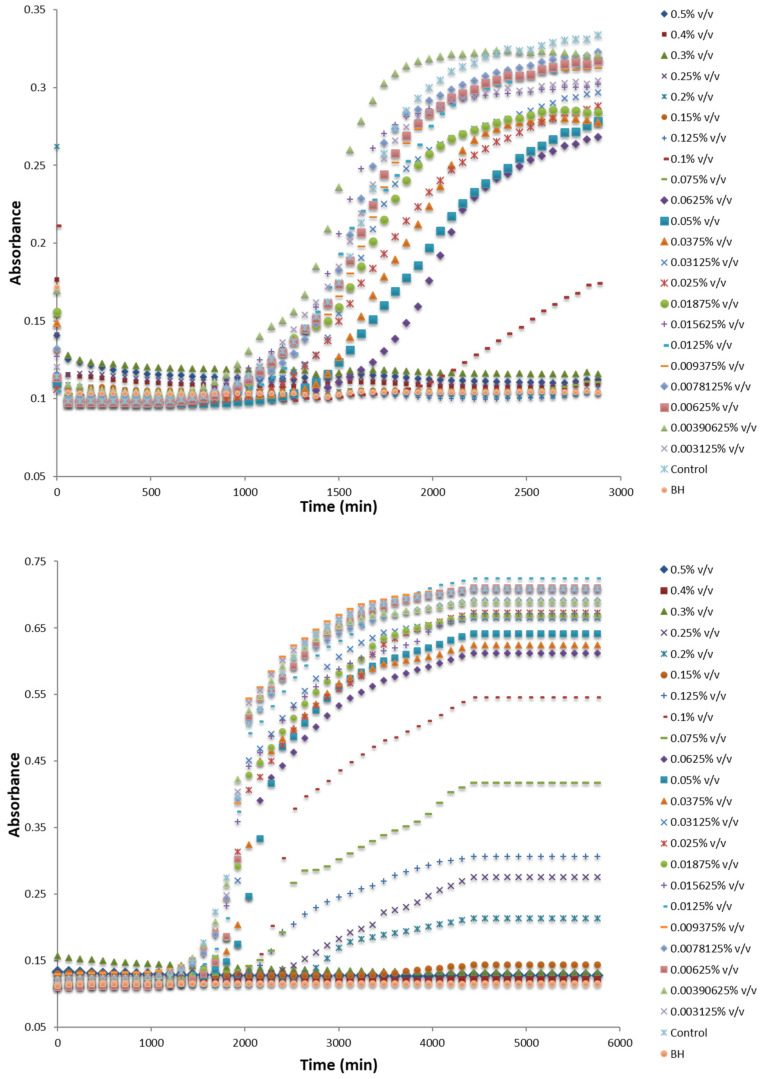
Typical growth curves of *Paenibacillus* sp. R2P2 (**upper diagram**) and *Penicillium* sp. R1R6 (**bottom diagram**) as observed by absorbance measurements at increasing concentrations from 0.003125% to 0.5% (*v*/*v*) of *Satureja thymbra* (**C**) essential oil in BHI and YES broth, respectively.

**Figure 2:**
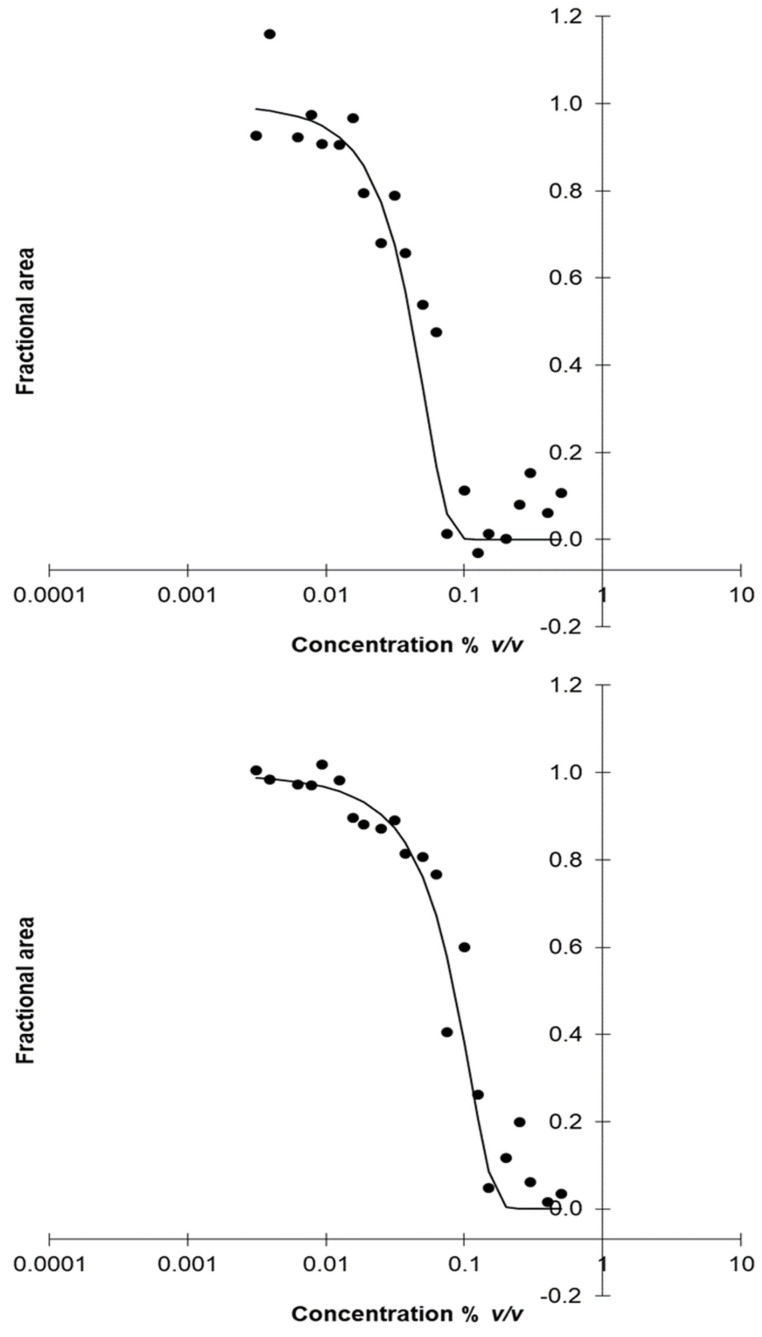
The inhibition profile of *Satureja thymbra* (C) essential oil against *Paenibacillus* sp. R2P2 (**upper diagram**) and *Penicillium* sp. R1R6 (**bottom diagram**) using absorbance measurements; (●) observed Fractional Area, (─) predicted Fractional Area.

Among the various methods used to assay the MIC of antimicrobials, a method developed by Lambert and Pearson [59] constitutes an automated technique that combines the OD measurements with the common dilution method [55,79]. This method has the advantage to allow the quick and efficient MIC assay and is applicable on single compounds and/or preservatives mixtures [81]. The accuracy of MIC determinations was ensured via the mathematical process of data [55,59,79]. More specifically, the areas under the OD/time curves were calculated using the trapezoidal rule (Figure 1) and the relative amount of growth—termed as fractional area, *fa*—was obtained as the ratio of the test area to that of control [55,59,79]. The plot of the inhibitory concentration with the fractional area (on a logarithmic scale) gave a characteristic sigmoid-shaped curve (Figure 2). As MIC was assigned the concentration above which no growth is observed relative to the control, while as NIC was defined the concentration below which normal visible growth was observed [55,59,79].

The experimental data were modeled using Equation (1), while the MIC and NIC values were calculated from Equations (2) and (3), respectively. The obtained MIC and NIC data of the representative bacterial (*Bacillus* sp. R1P4, *Stenotrophomonas* sp. S2P9, *Paenibacillus* sp. R2P2 and *Paenibacillus* sp. BP6) and fungal (*Fusarium* sp. R2R11, *Penicillium* sp. R1R6, *Clonostachys* sp. S1R1 and *Cladosporium* sp. BP1) isolates have been summarized in Table 5. In general, according to the results for the bacterial strains tested, the lower MIC and NIC values were observed for the EO of *Origanum vulgare* wild (Q) in comparison with the other one oregano EO (R). In this respect, the MIC values for *Bacillus* sp. R1P4 and *Stenotrophomonas* sp. S2P9 for *Origanum vulgare* wild (Q), were 0.015% *v*/*v* and 0.016% *v*/*v*, respectively, while for *Origanum vulgare* (R) were 0.069% *v*/*v* and 0.071% *v*/*v*, respectively and for *Satureja thymbra* (C) were 0.135% *v*/*v* and 0.157% *v*/*v*, respectively (Table 5). Regarding the bacterial strains *Paenibacillus* sp. R2P2 and *Paenibacillus sp.* BP6, similar MIC values were observed for *O. vulgare* wild (Q) and *O. vulgare* (R), while higher MIC values were noticed for *Satureja thymbra* (C) (0.074% *v*/*v* and 0.152% *v*/*v*, for *Paenibacillus* sp. R2P2 and *Paenibacillus* sp. BP6, respectively) (Table 5).

The results differed in terms of fungi. The minimum MIC value of 0.013% *v*/*v* was detected for *Fusarium* sp. R2R11, for *Satureja thymbra* (C) essential oil, which was much lower compared to the other two tested essential oils of *Origanum vulgare* (R and Q), with the respected MIC values of 0.097% *v*/*v* and 0.079% *v*/*v* (Table 5). For *Penicillium* sp. R1R6, the lower MIC value was noticed for *Origanum vulgare* wild (Q) (0.083% *v*/*v*), followed by those of *Origanum vulgare* (R) (0,145% *v*/*v*) and *Satureja thymbra* (C) (0.156% *v*/*v*). Similar results were observed for fungal strains *Clonostachys* sp. S1R1 and *Cladosporium* sp. BP1.

According to the literature, *Satureja* and *Origanum* EOs display superior antimicrobial activities, which may be attributed to the presence of monoterpene phenols, as well as to the secondary and synergistic effect of biologically active minor constituents, such as *γ*−terpinene and *p*-cymene [80,81,82,83,84]. It has been reported, that carvacrol and thymol were the monoterpene phenols that influenced, to the highest degree, the antibacterial action of several Greek EOs [51]. Thus, the chemical content indicated that the antibacterial properties of those studied EOs greatly depended on their content of the monoterpene phenols, carvacrol and thymol [51]. Additionally, research findings on EOs of Greek *Origanum* and *Satureja* plants have shown that the sum of these monoterpenes always represents the bulk of their EOs, regardless of their cultivation site and/or harvesting time [51,85]. In this respect, the presented MICs in the literature regarding *Origanum* and *Satureja* EOs, are always similar among respective microorganisms and with significant effect [55,79,85].

The application of a variety of conventional biocides in show caves has been previously explored. Martin-Sanchez et al. [86] have investigated the effect of biocides’ combination on the Lascaux cave over the years. They have shown that the biocides application increased the fungal biodiversity, but also noted that some microorganisms that were found in the cave (*Ochroconis* spp., *Acremonium nepalense* and the members of Herpotrichiellaceae) can metabolize a variety of organic carbon nutrients containing lignin phenols and alkylbenzenes, and their specialization in the selective degradation of these structures could explain their abundance.

In another study, Mitova et al. [15] have evaluated the susceptibility of several fungal isolates from Magura cave to conventional biocides i.e., BAC and OIT in concentrations of 100, 500, and 1000 mg/L, following lower concentrations than the manufacturers’ instructions (1 to 15 g/L). The susceptibility of the different isolates varied, with OIT exhibiting higher inhibition zones in high concentrations. However, in some strains, the inhibition zones did not vary between the different concentrations applied, giving thus no clear indication about the preferred concentration. In a similar way, the effect of thymol, silver nitrate and copper sulphate were tested in different concentrations (0.1%, 0.5% and 1.0%) against bacterial isolates from Magura cave [38]. Although the silver nitrate and copper sulphate showed antimicrobial activity in lower concentrations, the authors proposed thymol (with 1.0% exhibiting the highest antimicrobial activity) since this compound is transparent, colourless and did not damage additionally the cave paintings.

Previous studies have evaluated the antimicrobial activity of essential oils or their compounds against microorganisms on over-ground cultural heritage objects [39,40], mural paintings [41,42,43] or mosaic tesserae [44]. In detail, Stupar et al. [39], in a similar approach with the current study, applied the microdilution method to define the MIC of *O. vulgare, Rosmarinus officinalis* and *Lavantula angustifolia* EOs in comparison with the conventional BAC on five fungal strains isolated from cultural heritage objects [39]. It was also demonstrated that the oregano EO had the greatest antimicrobial activity with MIC values ranging between 0.2–0.5 μL/mL (i.e., 0.02–0.05% *v*/*v*), while the other EOs showed low antimicrobial activity. The corresponding MIC values for the conventional biocide applied (BAC) were 0.1–0.25μL/mL (i.e., 0.01–0.025% *v*/*v*). In another study, the EOs of *Pimpinella anisum, Syzygium aromaticum, Cuminum cyminum, Allium sativum, L. nobilis, C. sinensis, Osbeck and O. vulgare* were studied for their action against 4 fungal and 6 bacterial strains isolated from Cuban and Argentine Documentary Heritage, using the hole-plate diffusion method and relatively high concentrations (7.5–100%). *O. vulgare* EO was not only reported to be effective against all targets but additionally prevented fungal sporulation [40].

Veneranda et al. [42] have evaluated the biocide activity of *O. vulgare* constituents against a wild strain of *Aspergillus niger* isolated from a mural painting in Pompeii (Italy) using the agar disc diffusion assay and concentrations between 0.1–100% (*w*/*w*). It was demonstrated that thymol and eugenol showed an inhibition effect after incubation for up to 20 days at 1% and up to 30 days at 10%. Sakr et al. [41] studied the efficacy of five EOs (lemon, spearmint, fennel, marjoram and rosemary) against 4 yeast strains colonizing mural paintings in Ancient Egyptian tombs, by applying the agar disc diffusion assay with pure EOs (100%). Spearmint was found to be the only effective oil against all the tested strains. Marco et al. [43] have studied different essential oils (*R. officinalis, Foeniculum vulgare, C. limon, Ocimum basilicum* and *S. officinalis*) in parallel with commercial biocides (using the serial agar dilution method), to evaluate in vitro their efficacy against 4 fungal isolates from mural paintings. They have demonstrated that basil EO was the most effective against all the tested strains, able to inhibit their growth at 1.25 μL/mL (i.e., 0.125% *v*/*v*). Finally, Rotolo et al. [44] have evaluated two EOs (*Thymus vulgaris and O. vulgare* preliminarily in vitro (agar disc diffusion method) and after *ex situ* and *in situ* to control the biofilm (bacteria, fungi, cyanobacteria and green algae) colonization on the mosaic tesserae in the Greco-Roman archeological site of Solunto, Sicily (Italy). *T. vulgaris* solution was proven to be the best diffused, strongly influencing the biofilm liveliness, however the tested concentration was high (15% EO).

It has to be noted that the effectiveness of the selected EOs studied in this work has to be evaluated in situ, by applying the final product in real cave ecosystems. This final product is currently being developed and contains the selected EOs and the required environmentally friendly emulsifiers, stabilizers etc. with spraying the cave walls being the main application method. The effectiveness of the new product is important to be tested throughout the years, while evaluating the fragile microbial ecosystem of caves in parallel is of great interest. A characteristic example of the how a biocide may effect a cave ecosystem is the Lascaux cave. In this case, the serial treatments with biocides and antibiotics, eliminated the biocide-sensitive microbiota and promoted the growth of resistant microorganisms, resulting in developments of wall stains, i.e., green stains, later on white stains and more recently black stains [25]. Another issue that is crucial for the biocide effectiveness is the presence of microbial biofilms. Development of biofilms, built from extracellular polymeric substances (e.g., polysaccharides, lipids, proteins, nucleic acids, pigments, enzymes), may significantly enhance the biodeterioration processes offering in parallel increased resistance of microorganisms to biocidal compounds [87]. All the aforementioned matters have to be closely monitored irrespectively of the biocide origin (natural or not) and according to the findings adjust the application frequency and maybe rotate the use of different EOs.

## 4. Conclusions

In the current study, 18 essential oils (EO) of plant origin extracted from various Greek plants were evaluated for their antimicrobial activity against 35 bacterial and 31 fungi isolates (isolated from a Greek cave). It was shown that 3 EO (2 *Origanum vulgare* and 1 *Satureja thymbra*) exhibited high antimicrobial activity against all the tested microbiota, in low concentrations (MIC < 0.16 % *v*/*v*) which constitutes their commercial exploitation sustainable. Thus, these EO have the potential to be applied as herbal biocides to address the serious issue of interior alteration observed in show caves due to the development of various microorganisms. In this direction, future studies are needed to confirm the findings of this study in a real cave ecosystem.

## Figures and Tables

**Table 1:** Collection data of tested essential oils.

Code	Essential Oil	Locality	Part Distilled	Ref. ^1^
A	*Salvia triloba*	Rethymno, Creta	Aerial parts	[51]
B	*Foeniculum vulgare*	Astros, Peloponnese	Aerial parts	[52]
C	*Satureja thymbra*	Parnonas, Peloponnese	Aerial parts	[52]
D	*Juniperus phoenicea*	Antikyra, Central Greece	Berry reap crushed	[54]
E	*Juniperus drupacea*	Antikyra, Central Greece	Berry reap crushed	[54]
F	*Juniperus drupacea*	Antikyra, Central Greece	Berry reap crushed	[54]
G	*Citrus x paradisii*	Argolida, Peloponnese	By-products during Fruit liquidization	[53]
H	*Citrus x paradisii*	Argolida, Peloponnese	By-products during Fruit liquidization-Fragment 1	[53]
I	*Citrus limon*	Argolida, Peloponnese	By-products during Fruit liquidization	[53]
J	*Citrus reticulata*	Argolida, Peloponnese	By-products during Fruit liquidization	[53]
K	*Citrus reticulata*	Argolida, Peloponnese	By-products during Fruit liquidization-Fragment 1	[53]
L	*Citrus sinensis*	Argolida, Peloponnese	By-products during Fruit liquidization	[53]
M	*Juniperus phoenicea*	Antikyra, Central Greece	Berry reap crushed	[54]
N	*Citrus limon*	Corinthos, Peloponnese	Fruit Unreap	[53]
O	*Citrus aurantium*	Corinthos, Peloponnese	Fruit Reap	[53]
P	*Laurus nobilis*	Chania, Creta	Leaves	[51]
Q	*Origanum vulgare* wild	Kozani, North Greece	Aerial parts	[51]
R	*Origanum vulgare*	Kilkis, North Greece	Aerial parts	[52]

^1^ Obtained according to the corresponding reference.

**Table 2:** Antimicrobial activity of the 18 EOs on the bacterial isolates.

Isolate	Isolate with the Same Phenotype	Essential oil ( Inhibitory Concentration % *v/v*)																	
		A	B	C	D	E	F	G	H	I	J	K	L	M	N	O	P	Q	R
*Bacillus* sp. R1P2		5.0	5.0	0.1	5.0	5.0	5.0	5.0	5.0	5.0	5.0	5.0	5.0	5.0	5.0	5.0	5.0	0.1	0.1
*Bacillus mycoides* R1P3		5.0	5.0	0.1	5.0	5.0	5.0	5.0	5.0	5.0	5.0	5.0	5.0	5.0	5.0	5.0	5.0	0.1	0.1
*Bacillus* sp. R1P4		5.0	5.0	0.1	5.0	5.0	5.0	5.0	5.0	5.0	5.0	5.0	5.0	5.0	5.0	5.0	5.0	0.1	0.1
*Bacillus thuringiensis* R1P5		5.0	5.0	0.1	5.0	5.0	5.0	5.0	5.0	5.0	5.0	5.0	5.0	5.0	5.0	5.0	5.0	0.1	0.1
*Bacillus* sp. R1P6	R1P8, R1P9, R1P10	5.0	5.0	0.1	5.0	5.0	5.0	5.0	5.0	5.0	5.0	5.0	5.0	5.0	5.0	5.0	5.0	0.1	0.1
*Bacillus* sp. R2P1		5.0	5.0	0.1	5.0	5.0	5.0	5.0	5.0	5.0	5.0	5.0	5.0	5.0	5.0	5.0	1.0	0.1	0.1
*Bacillus* sp. R2P3		5.0	5.0	0.1	1.0	1.0	1.0	1.0	5.0	1.0	1.0	5.0	1.0	1.0	1.0	1.0	0.5	0.1	0.1
*Bacillus* sp. R2P4		5.0	5.0	0.1	5.0	5.0	5.0	5.0	5.0	5.0	5.0	5.0	5.0	5.0	5.0	5.0	5.0	0.1	0.1
*Bacillus* sp. R2P5		5.0	5.0	0.1	5.0	5.0	5.0	5.0	5.0	5.0	5.0	5.0	5.0	5.0	5.0	5.0	1.0	0.1	0.1
*Achromobacter* sp. R1R18		5.0	5.0	0.1	5.0	5.0	5.0	5.0	5.0	5.0	5.0	5.0	5.0	5.0	5.0	5.0	5.0	0.1	0.1
*Achromobacter* sp. R1R21		5.0	5.0	0.1	5.0	5.0	5.0	5.0	5.0	5.0	5.0	5.0	5.0	5.0	1.0	5.0	5.0	0.1	0.1
*Achromobacter* sp. R1P1		5.0	5.0	0.1	5.0	5.0	5.0	5.0	5.0	5.0	5.0	5.0	5.0	5.0	1.0	5.0	5.0	0.1	0.1
*Achromobacter* sp. S1P1		5.0	1.0	0.1	5.0	5.0	5.0	5.0	5.0	5.0	5.0	5.0	5.0	5.0	1.0	5.0	1.0	0.1	0.1
*Achromobacter* sp. S1P3		5.0	5.0	0.1	5.0	5.0	5.0	5.0	5.0	1.0	5.0	5.0	5.0	5.0	1.0	5.0	1.0	0.1	0.1
*Achromobacter* sp. BP3		5.0	5.0	0.1	5.0	5.0	5.0	5.0	5.0	5.0	5.0	5.0	5.0	5.0	5.0	5.0	5.0	0.1	0.1
*Achromobacter* sp. S2P2		5.0	5.0	0.1	5.0	5.0	5.0	5.0	5.0	5.0	5.0	5.0	5.0	5.0	1.0	5.0	5.0	0.1	0.1
*Achromobacter* sp. S2P3		5.0	5.0	0.1	5.0	5.0	5.0	5.0	5.0	5.0	5.0	5.0	5.0	5.0	1.0	5.0	1.0	0.1	0.1
*Achromobacter* sp. S2P4		5.0	1.0	0.1	5.0	5.0	5.0	5.0	5.0	5.0	5.0	5.0	5.0	5.0	1.0	5.0	5.0	0.1	0.1
*Sinorhizobium* (*Ensifer*) *adhaerens* R1R20		5.0	5.0	0.1	5.0	5.0	5.0	5.0	5.0	5.0	5.0	5.0	5.0	5.0	1.0	5.0	0.5	0.1	0.1
*Sinorhizobium (Ensifer) adhaerens* R1R22		5.0	5.0	0.1	5.0	5.0	5.0	5.0	5.0	5.0	5.0	5.0	5.0	5.0	1.0	5.0	1.0	0.1	0.1
*Sinorhizobium* sp. R2R10		5.0	1.0	0.1	5.0	5.0	5.0	5.0	5.0	5.0	5.0	5.0	5.0	5.0	1.0	5.0	1.0	0.1	0.1
*Sinorhizobium* sp. BP4		5.0	5.0	0.1	5.0	5.0	5.0	5.0	5.0	5.0	5.0	5.0	5.0	5.0	1.0	5.0	0.5	0.1	0.1
*Paenibacillus* sp./*wynii/graminis* S2P5		5.0	5.0	0.1	1.0	5.0	1.0	1.0	5.0	1.0	1.0	5.0	5.0	1.0	1.0	1.0	0.5	0.1	0.1
*Paenibacillus* sp. R2P2		5.0	5.0	0.1	5.0	5.0	5.0	1.0	1.0	1.0	5.0	5.0	5.0	1.0	5.0	1.0	0.5	0.1	0.1
*Paenibacillus amylolyticus* BP5		5.0	5.0	0.1	1.0	1.0	1.0	1.0	5.0	1.0	1.0	5.0	1.0	1.0	1.0	1.0	0.5	0.1	0.1
*Paenibacillus* sp. BP6		5.0	5.0	0.1	5.0	5.0	1.0	1.0	5.0	1.0	1.0	5.0	1.0	1.0	5.0	1.0	0.5	0.1	0.1
*Rhodococcus erythropolis* S1P2		5.0	5.0	0.1	5.0	5.0	5.0	5.0	5.0	5.0	5.0	5.0	5.0	5.0	5.0	5.0	1.0	0.1	0.1
*Rhodococcus* sp. S2P6		5.0	5.0	0.1	5.0	5.0	5.0	5.0	5.0	5.0	5.0	5.0	5.0	5.0	1.0	5.0	1.0	0.1	0.1
*Rhodococcus* sp. S2P7		5.0	5.0	0.1	5.0	5.0	5.0	5.0	5.0	5.0	5.0	5.0	5.0	5.0	5.0	5.0	1.0	0.1	0.1
*Rhodococcus* sp. S2P8		5.0	5.0	0.1	5.0	5.0	5.0	5.0	5.0	5.0	5.0	5.0	5.0	5.0	5.0	5.0	1.0	0.1	0.1
*Stenotrophomonas* sp. S1P4		5.0	5.0	0.1	5.0	5.0	5.0	5.0	5.0	5.0	5.0	5.0	5.0	5.0	5.0	5.0	5.0	0.1	0.1
*Stenotrophomonas* sp. S2P9		5.0	5.0	0.1	5.0	5.0	5.0	5.0	5.0	5.0	5.0	5.0	5.0	5.0	1.0	5.0	1.0	0.1	0.1

A-Salvia triloba, B-Foeniculum vulgare, C-Satureja thymbra, D-Juniperus phoenicea, E-Juniperus drupacea, F-Juniperus drupacea, G-Citrus x paradisii, H-Citrus x paradisii, I-Citrus limon, J-Citrus reticulata, K-Citrus reticulate, L-Citrus sinensis, M-Citrus sinensis, N-Citrus limon, O-Citrus aurantium, P-Laurus nobilis, Q-Origanum vulgare, R-Origanum vulgare. Isolate coding: R_n_R_n_ isolated from weathered rocks on RBC, R_n_P_n_ isolated from weathered rocks on PCA, S_n_P_n_, isolated from swab streaking on PCA, BP_n_ isolated from swab supernatant on PCA. Isolate with the same phenotype: based on oxidase and catalase reaction, colony morphology, Gram stain and cell morphology, resistance to the EOs tested. Bold numbers indicate the lower inhibitory concentration.

**Table 3:** Antimicrobial activity of the 18 EOs on the fungal isolates.

Isolate	Isolate with the Same Phenotype	Essential Oil ( Inhibitory Concentration % *v/v*)																	
		A	B	C	D	E	F	G	H	I	J	K	L	M	N	O	P	Q	R
*Penicillium vulpinum* R1R3		5.0	5.0	0.2	5.0	5.0	5.0	5.0	5.0	5.0	5.0	5.0	5.0	5.0	5.0	5.0	0.5	0.1	0.1
*Penicillium* sp. R1R5	R1R11, R1R12	5.0	5.0	0.2	5.0	5.0	5.0	5.0	5.0	5.0	5.0	5.0	5.0	5.0	5.0	5.0	0.5	0.1	0.1
*Penicillium* sp. R1R6		5.0	5.0	0.5	5.0	5.0	5.0	5.0	5.0	5.0	5.0	5.0	5.0	5.0	5.0	5.0	5.0	0.1	0.1
*Penicillium* sp. S1R5		5.0	5.0	0.2	5.0	5.0	5.0	5.0	5.0	5.0	5.0	5.0	5.0	5.0	5.0	1.0	0.5	0.1	0.1
*Penicillium* sp. S1R6	S1R2	1.0	1.0	0.1	5.0	5.0	5.0	5.0	5.0	5.0	5.0	5.0	5.0	5.0	5.0	5.0	5.0	0.1	0.1
*Penicillium* sp. S2R1		1.0	1.0	0.1	5.0	5.0	5.0	5.0	5.0	5.0	5.0	5.0	5.0	5.0	1.0	5.0	0.5	0.1	0.1
*Clonostachys* sp. R1R7	R1R1, R1R4, R1R8	5.0	5.0	0.2	5.0	5.0	5.0	5.0	5.0	5.0	5.0	5.0	5.0	5.0	1.0	5.0	5.0	0.1	0.1
*Clonostachys* sp. S1R1		5.0	5.0	0.2	5.0	5.0	5.0	5.0	5.0	5.0	5.0	5.0	5.0	5.0	1.0	5.0	0.5	0.1	0.1
*Fusarium solani* R2R5		5.0	5.0	0.5	5.0	5.0	5.0	5.0	5.0	5.0	5.0	5.0	5.0	5.0	5.0	5.0	0.5	0.1	0.1
*Fusarium solani* R2R8	R2R4	5.0	5.0	0.5	5.0	5.0	5.0	5.0	5.0	5.0	5.0	5.0	5.0	5.0	5.0	5.0	5.0	0.1	0.1
*Fusarium* sp. R2R11		5.0	5.0	0.5	5.0	5.0	5.0	5.0	5.0	5.0	5.0	5.0	5.0	5.0	5.0	5.0	5.0	0.1	0.1
*Doratomyces stemonitis* R2R3	R2R12, R2R14, R2S2	5.0	5.0	0.2	5.0	5.0	5.0	5.0	5.0	5.0	5.0	1.0	5.0	5.0	1.0	5.0	0.5	0.1	0.1
*Cephalotrichum verrucisporum*/*oligotriphicum* R2R1		1.0	1.0	0.1	5.0	1.0	5.0	5.0	5.0	5.0	5.0	5.0	5.0	5.0	1.0	1.0	0.5	0.1	0.1
*Cephalotrichum* sp. R1R2	R1R9	1.0	1.0	0.1	5.0	5.0	1.0	5.0	5.0	5.0	5.0	1.0	5.0	5.0	1.0	1.0	0.5	0.1	0.1
*Talaromyces minioluteus* R2R7	R2R2	5.0	5.0	0.2	5.0	5.0	5.0	5.0	5.0	5.0	5.0	5.0	5.0	5.0	5.0	5.0	0.5	0.1	0.1
*Acremonium persicinum* R2S1		1.0	5.0	0.2	5.0	5.0	1.0	5.0	1.0	5.0	5.0	1.0	5.0	5.0	1.0	1.0	0.5	0.1	0.1
*Xenoacremonium falcatus* R1R15		5.0	5.0	0.1	5.0	5.0	5.0	5.0	5.0	5.0	5.0	5.0	5.0	5.0	1.0	5.0	5.0	0.1	0.1
*Trichurus* sp. R1S1		5.0	5.0	0.2	5.0	5.0	5.0	5.0	5.0	5.0	5.0	5.0	5.0	5.0	5.0	5.0	5.0	0.1	0.1
*Cladosporium* sp. BP1		5.0	5.0	0.1	5.0	5.0	5.0	5.0	5.0	5.0	5.0	1.0	5.0	5.0	1.0	1.0	0.5	0.1	0.1

A-Salvia triloba, B-Foeniculum vulgare, C-Satureja thymbra, D-Juniperus phoenicea, E-Juniperus drupacea, F-Juniperus drupacea, G-Citrus x paradisii, H-Citrus x paradisii, I-Citrus limon, J-Citrus reticulata, K-Citrus reticulate, L-Citrus sinensis, M-Citrus sinensis, N-Citrus limon, O-Citrus aurantium, P-Laurus nobilis, Q-Origanum vulgare, R-Origanum vulgare. Isolate coding: R_n_R_n_, R_n_S_n_, isolated from weathered rocks on RBC, S_n_R_n_ isolated from swab streaking on RBC, BP_n_ isolated from swab supernatant on PCA. Isolate with the same phenotype: Isolates with the same colony morphology on the different growth media and same resistance to the EOs tested. Bold numbers indicate the lower inhibitory concentration.

**Table 4:** Total number of bacterial and fungal isolates inhibited by the 18 EOs in concentrations up to 1% (*v*/*v*).

Essential Oil		Inhibitory Concentration (% *v*/*v*)	Growth Inhibition (No of Isolates)		
			Fungi (*n* = 31)	Bacteria (*n* = 35)	Total (*n* = 66)
A	*Salvia triloba*	1.0	7	0	7
B	*Foeniculum vulgare*	1.0	6	3	9
C	*Satureja thymbra*	0.1	8	35	43
		0.2	18	0	18
		0.5	5	0	5
		*Total*	31	35	66
D	*Juniperus phoenicea*	1.0	0	3	3
E	*Juniperus drupacea*	1.0	1	2	3
F	*Juniperus drupacea*	1.0	3	4	7
G	*Citrus x paradisii*	1.0	0	5	5
H	*Citrus x paradisii*	1.0	1	1	2
I	*Citrus limon*	1.0	0	6	6
J	*Citrus reticulate*	1.0	0	4	4
K	*Citrus reticulate*	1.0	8	0	8
L	*Citrus sinensis*	1.0	0	3	3
M	*Citrus sinensis*	1.0	0	5	5
N	*Citrus limon*	1.0	16	16	32
O	*Citrus aurantium*	1.0	6	5	11
P	*Laurus nobilis*	0.5	19	7	26
		1.0	0	12	12
		*Total*	19	19	38
Q	*Origanum vulgare* (wild)	0.1	31	35	66
R	*Origanum vulgare*	0.1	31	35	66

**Table 5:** MIC and NIC values and their standard errors (% *v*/*v* concentration of Origanum vulgare, Satureja thymbra, *Origanum vulgare* wild essential oils in BHI broth for the bacteria and in YES broth for the fungi).

	*Origanum vulgare* (R)		*Satureja thymbra* (C)		*Origanum vulgare* wild (Q)	
	MIC	NIC	MIC	NIC	MIC	NIC
*Bacillus* sp. RIP4	0.069 ± 0.002	0.053 ± 0.001	0.135 ± 0.003	0.091 ± 0.001	0.015 ± 0.004	0.005 ± 0.002
*Stenotrophomonas* sp. S2P9	0.071 ± 0.003	0.016 ± 0.004	0.157 ± 0.002	0.038 ± 0.001	0.016 ± 0.001	0.004 ± 0.001
*Paenibacillus* sp. R2P2	0.041 ± 0.001	0.009 ± 0.001	0.074 ± 0.002	0.021 ± 0.001	0.039 ± 0.003	0.018 ± 0.002
*Paenibacillus* sp. BP6	0.051 ± 0.003	0.028 ± 0.002	0.152 ± 0.003	0.069 ± 0.002	0.088 ± 0.002	0.032 ± 0.001
*Fusarium* sp. R2R11	0.097 ± 0.010	0.027 ± 0.007	0.013 ± 0.010	0.027 ± 0.007	0.079 ± 0.009	0.016 ± 0.005
*Penicillium* sp. R1R6	0.145 ± 0.009	0.035 ± 0.006	0.156 ± 0.008	0.038 ± 0.008	0.083 ± 0.008	0.018 ± 0.003
*Clonostachys* sp. S1R1	0.103 ± 0.008	0.031 ± 0.009	0.106 ± 0.009	0.028 ± 0.004	0.072 ± 0.010	0.016 ± 0.005
*Cladosporium* sp. BP1	0.101 ± 0.011	0.038 ± 0.008	0.064 ± 0.007	0.017 ± 0.005	0.054 ± 0.011	0.015 ± 0.002

## Data Availability

All additional data can be obtained from the corresponding authors upon reasonable request.

## Supplementary Materials

The following are available online at https://www.mdpi.com/article/10.3390/microorganisms9091836/s1, Table S1: Identity of bacteria isolated from Petralona Cave, Table S2: Identity of fungi isolated from Petralona Cave.
