# Supplementary material for: Evaluation of Plant Origin Essential Oils as Herbal Biocides for the Protection of Caves Belonging to Natural and Cultural Heritage Sites
Source: Microorganisms. 2021 Aug 30;9(9):1836. doi: 10.3390/microorganisms9091836 (PMC8470480; doi:10.3390/microorganisms9091836)
Supplement: Supplementary file 1 [file microorganisms-09-01836-s001.zip › microorganisms-1310875-supplementary.pdf]

microorganisms

MDPI

Supplementary Table S1. Identity of bacteria isolated from Petralona Cave

Isolate	Closest relative species (Accession number)	Similarity (%)
Bacillus sp. R1P2	*Bacillus* sp. strain YMA_01 (MT360214)	99.83
Bacillus mycoides R1P3	*Bacillus mycoides* strain BPN51/1 (CP036017)	100.00
Bacillus sp. R1P4	*Bacillus* sp. strain YMA_01 (MT360214)	99.84
Bacillus thuringiensis R1P5	*Bacillus thuringiensis* strain SBMG3 (MZ314373)	99.51
Bacillus sp. R1P6	*Bacillus* sp. strain C3_RS_20 (MT354350)	99.84
Bacillus sp. R2P1	*Bacillus* sp. strain C2NPK_RS_14 (MT354274)	99.83
Bacillus sp. R2P3	*Bacillus* sp. strain MAIDO-R2b-3 (MW711674)	99.53
Bacillus sp. R2P4	*Bacillus* sp. strain UFSC-20S3 (MT269035)	99.67
Bacillus sp. R2P5	*Bacillus* sp. strain PBS32 (MW812285)	99.83
Achromobacter sp. R1R18	*Achromobacter* sp. strain T5712 (MT568550)	99.80
Achromobacter sp. R1R21	*Achromobacter* sp. YAZ103 (LC529453)	99.50
Achromobacter sp. R1P1	*Achromobacter* sp. strain T5712 (MT568550)	99.34
Achromobacter sp. S1P1	*Achromobacter* sp. strain Bel (MT299752)	99.83
Achromobacter sp. S1P3	*Achromobacter* sp. strain Bel (MT299752)	99.83
Achromobacter sp. BP3	*Achromobacter* sp. strain T5712 (MT568550)	99.83
Achromobacter sp. S2P2	*Achromobacter* sp. YAZ43 (LC529453)	99.83
Achromobacter sp. S2P3	*Achromobacter* sp. Ho12b (LC529439)	100.00
Achromobacter sp. S2P4	*Achromobacter* sp. NGB-WS19 (LC592758)	100.00
Sinorhizobium (*Ensifer*) *adhaerens* R1R20	*Ensifer adhaerens* strain RPB12 (MT772191)	100.00
Sinorhizobium (*Ensifer*) *adhaerens* R1R22	*Ensifer adhaerens* strain AS7B15 (MT640312)	100.00
Sinorhizobium sp. R2R10	*Ensifer adhaerens* strain AS7B15 (MT640312)	98.52
Sinorhizobium sp. BP4	*Ensifer adhaerens* strain AS7B15 (MT640312)	99.64

Paenibacillus sp. /*wynii*/ *graminis* S2P5	*Paenibacillus sp.* TmMRS5 (AB449074)/ *Paenibacillus wynnii* strain Hgh02-S4 (JQ436909)/ *Paenibacillus graminis* strain BJC15-B14 (JX401460)	98.80
Paenibacillus sp. R2P2	*Paenibacillus* sp. TB2019 (CP070969)	97.37
Paenibacillus amylolyticus BP5	*Paenibacillus amylolyticus* isolate as22b (AM062689)	99.67
Paenibacillus sp. BP6	*Paenibacillus* sp. strain ALG50H8 (MG775187)	99.83
Rhodococcus erythropolis S1P2	*Rhodococcus erythropolis* strain RYA9 (MT549100)	99.82
Rhodococcus sp. S2P6	*Rhodococcus* sp. strain NRCB009 (MN128369)	100.00
Rhodococcus sp. S2P7	*Rhodococcus* sp. strain NRCB009 (MN128369)	100.00
Rhodococcus sp. S2P8	*Rhodococcus* sp. strain YF-9 (MT631993)	99.09
Stenotrophomonas sp. S1P4	*Stenotrophomonas* sp. strain TP102 (MT764926)	100.00
Stenotrophomonas sp. S2P9	*Stenotrophomonas* sp. strain Rh1	99.66

Isolate coding: R_nR_n isolated from weathered rocks on RBC, R_nP_n isolated from weathered rocks on PCA, S_nP_n, isolated from swab streaking on PCA, BP_n isolated from swab supernatant on PCA.

Supplementary Table S2. Identity of fungi isolated from Petralona Cave

**Isolate**	**Closest relative species (Accession number)**	**Similarity (%)**
Penicillium vulpinum R1R3	*Penicillium vulpinum* strain CBS 140.45 (MH856133)	99.73
Penicillium sp. R1R5	*Penicillium* sp. GZU-BCECA56- 2 (GU565118)	98.87
Penicillium sp. R1R6	*Penicillium sp.* isolate CY03 (MW534771)	100.00
Penicillium sp. S1R5	*Penicillium* sp. (MT682669)	99.05
Penicillium sp. S1R6	*Penicillium* sp. isolate Fauno6A (MW881071)	99.78
Penicillium sp. S2R1	*Penicillium* sp. 19VA03 (JX270544)	98.06
Clonostachys sp. R1R7	*Clonostachys* sp. JCM 28116 (LC145304)	99.39
Clonostachys sp. S1R1	*Clonostachys sp.* isolate 18KTF1 (MT588112)	99.39
Fusarium solani R2R5	*Fusarium solani* strain R22 (MF687679.1)	100.00
Fusarium solani R2R8	*Fusarium solani* isolate MQ (MH300490)	98.24
Fusarium sp. R2R11	*Fusarium* sp. isolate BK230 (KU702686.1)	99.19
Doratomyces stemonitis R2R3	*Doratomyces stemonitis* strain 0909CI13LL_1 (FR799473)	99.63
Cephalotrichum verrucisporum / *oligotriphicum* R2R1	*Doratomyces verrucisporus* strain A31 (KX058039) /*Cephalotrichum oligotriphicum* strain LC7455 (MF419789)	98.90
Cephalotrichum sp R1R2	*Cephalotrichum* sp. LM-2019e isolate HHAUF170564 (MG886314)	98.03
Talaromyces minioluteus R2R7	*Penicillium minioluteum* strain E3 (GU566240)	97.57
Acremonium persicinum R2S1	*Acremonium persicinum* isolate PAV-M 1.147 (KF993390)	99.01
Xenoacremonium falcatus R1R15	*Xenoacremonium falcatus* (LT799728)	99.37
Trichurus sp. R1S1	*Trichurus* sp. 0109CI22V1 (FN598956)	100.00
Cladosporium sp. BP1	*Cladosporium sp.* isolate RL676 (MT557210.1)	98.15

Isolate coding: R_nR_n, R_nS_n, isolated from weathered rocks on RBC, S_nR_n isolated from swab streaking on RBC, BP_n isolated from swab supernatant on PCA.
